# Efficacy and safety of Kunxian in IgA nephropathy

**DOI:** 10.3389/fphar.2025.1496967

**Published:** 2025-03-04

**Authors:** Yang Yang, Xiang Li, Honghong Zou, Manna Li, Li Wang, Kaiping Luo, Wenjun Yan, Yebei Li, Baoqin Zhou, Wenling Kang, Lijuan Wang, Shizhang Xu, Gaosi Xu

**Affiliations:** ^1^ Department of Nephrology, The Second Affiliated Hospital, Jiangxi Medical College, Nanchang University, Nanchang, China; ^2^ Jiangxi Key Laboratory of Molecular Medicine, The Second Affiliated Hospital of Nanchang University, Nanchang, China; ^3^ Department of Nephrology, Ganzhou People’s Hospital, Ganzhou, China; ^4^ Department of Nephrology, The First Affiliated Hospital of Gannan Medical University, Ganzhou, China; ^5^ Department of Nephrology, Xinyu People’s Hospital, Xinyu, China; ^6^ Department of Nephrology, Shangrao People’s Hospital, Shangrao, China; ^7^ Department of Nephrology, Yichun People’s Hospital, Yichun, China

**Keywords:** IgA nephropathy, tripterygium wilfordii hook F, KunXian, proteinuria, glomerular filtration rate

## Abstract

**Background:**

Kunxian (KX) has been reported to be effective in treating Immunoglobulin A nephropathy (IgAN) and autoimmune disorders, such as lupus nephritis, but there is a lack of controlled trial on its effectiveness and safety for treating IgAN.

**Methods:**

This multicenter, prospective cohort study was conducted with individuals aged 18–60 years with biopsy-confirmed primary IgAN, proteinuria greater than 0.75 g/d, and estimated glomerular filtration rate (eGFR) greater than 60 mL/min/1.73 m^2^. Patients were treated with KX or Mycophenolate mofetil (MMF) after receiving a stable dose of an angiotensin-converting-enzyme inhibitor or angiotensin-receptor blocker for at least 4 weeks.

**Results:**

67 patients were assigned to the KX group and 72 to the MMF group. The mean (standard deviation) eGFR was 87.75 (15.94) mL/min/1.73 m^2^, and the mean (standard deviation) proteinuria was 1.70 (0.74) g/d. Patients in the KX group had a greater reduction in proteinuria than those in the MMF group did. Complete remission occurred in 43 patients (64.2%) in the KX group and 37 patients (51.4%) in the MMF group (hazard ratio [HR] 0.612, 95% CI 0.385–0.972, *P* = 0.038). Overall response occurred in 59 participants (88.1%) in the KX group and 59 participants (81.9%) in MMF group (HR 0.658, 95% CI 0.447–0.970, *P* = 0.034). Adverse events were observed in 6 patients (8.9%) in the KX group and 5 patients (6.9%) in the MMF group with no significant difference.

**Conclusion:**

Compared with MMF, KX was safe and significantly decreased proteinuria in IgAN.

## 1 Introduction

Immunoglobulin A nephropathy (IgAN), characterized by the deposition of immunoglobulin A (IgA) in the glomerular mesangium (Stamellou et al., 2023), is the most common biopsy-proven primary glomerular disease in China (Qin et al., 2023; Li and Liu, 2004). It has been reported that nearly 30%–40% of patients can develop end-stage renal disease within 20–30 years (Stamellou et al., 2023). However, there is no disease-specific treatment available for IgAN.

*Tripterygium Wilfordii* Hook F (TwHF) is a traditional herb first recorded in the Compendium of Materia Medic (Jia et al., 2021), and has been widely used for many years in the treatment of rheumatic diseases and autoimmune disorders (Shan et al., 2023; Zhang et al., 2024). In China, TwHF has been used as an immunosuppressant to reduce proteinuria for more than 40 years (Chen et al., 2013; Le et al., 2022). A meta-analysis revealed that TwHF significantly reduced proteinuria while maintaining good renal function in IgAN patients (Chen et al., 2010).

Kunxian (KX) is a derivative of TwHF, which is mainly composed of four medicinal herbs, namely, Tripterygium hypoglaucum Hutch (triptolide), Epimedium brevicornu Maxim, Cuscuta chinensis Lam, and Lycium barbarum L (Ma et al., 2023). The incidence of adverse events (AEs) of KX was significantly lower than that of TwHF(Tang et al., 2020), which may be associated with the drug combination of herbal medicines. Several studies have indicated that the anti-inflammatory, immunomodulatory, and analgesic properties of KX demonstrate favorable efficacy in patients with ankylosing spondylitis and rheumatoid arthritis (Tang et al., 2020; Li et al., 2016). Evidence suggests that KX attenuates foot cell damage by inhibiting β-catenin signaling, thereby ameliorating proteinuria in diabetic mice without inducing significant hepatorenal toxicity being observed (Jin et al., 2023). Moreover, one study reported that 97% of IgAN patients experienced a reduction in proteinuria within 2 months after KX administration, and 45.5% of patients achieved a level of proteinuria less than 0.5 g/d at 28 weeks (Le et al., 2022). However, long-term controlled trials verifying the effectiveness and safety of KX for the treatment of IgAN are lacking.

## 2 Materials and methods

### 2.1 Study design and patients

The present multicenter, prospective cohort study was conducted at six research centers in China (the Second Affiliated Hospital of Nanchang University, the First Affiliated Hospital of Gannan Medical University, Ganzhou People’s Hospital, Xinyu People’s Hospital, Shangrao People’s Hospital, and Yichun People’s Hospital) from March 2018 to March 2020. The present study was approved by the Ethics Committee of the Second Affiliated Hospital of Nanchang University and all patients provided written informed consent.

All participants were required to receive a stable dose of an angiotensin-converting-enzyme inhibitor or angiotensin-receptor blocker for at least 4 weeks before grouping. Persons aged 18–60 years with biopsy-confirmed primary IgAN, urinary protein (UP) > 0.75 g/d, and an estimated glomerular filtration rate (eGFR) > 60 mL/min/1.73 m^2^ as estimated by the Chronic Kidney Disease Epidemiology Collaboration (Levey et al., 2009) were enrolled. Patients with the following conditions were excluded: any secondary form of IgAN or IgA vasculitis or any non-IgAN glomerulonephritis, major hepatic, cerebrovascular, or cardiovascular comorbidities, or prior kidney transplantation.

### 2.2 Intervention and follow-up

All eligible patients were assigned to receive KX (KX group) or mycophenolate mofetil (MMF group). Patients treated with KX were administered an initial dose of 0.8–1.2 g daily for 3 months, then tapered to a maintenance daily dose of 0.6–0.8 g for the subsequent 6 months. The subjects treated with MMF received an oral dose of 1.25–1.5 g daily for 6 months, followed by tapering to a maintenance dose of 0.75–1.0 g daily for 6 months. Throughout the study, participants were followed up at baseline, 1 month, 3 months, and then every 3 months thereafter.

### 2.3 Study outcome

The primary outcomes were complete remission (CR) and the rate of an overall response (OR). CR was defined as 24 h UP < 0.3 g, and OR was defined as CR plus partial remission (PR), with PR defined as UP 0.3–2 g/d, but with a reduction greater than 50% from baseline. The time to a 30% reduction in the eGFR was also evaluated. The secondary outcomes included urine occult blood, urine red blood cells (URBC), and any AEs.

### 2.4 Statistical analysis

The continuous variables were described as mean ± standard deviation (SD), and the categorical variables were presented as numbers and percentages. Intergroup comparisons were performed using the Pearson chi-square test or Fisher’s exact test and Mann-Whitney U-test. The variation in the eGFR and 24 h UP level during different periods were compared between the two groups of IgAN patients. Kaplan-Meier curve analysis was used to describe the time-to-event data, and the difference between the two groups were compared by the log-rank test. Survival time was determined from the beginning of the first treatment until the event of interest. Patients who did not achieve remission were considered censored at the time of the last visit. Treatment effects on the two primary outcomes were estimated using Cox proportional hazard models with covariate adjustment. All statistical analyses were conducted by SPSS (version 28.0) and GraphPad (version 9.0). Two-sided *P* < 0.05 was regarded as statistically different.

## 3 Results

### 3.1 Baseline characteristics

**FIGURE 1:**
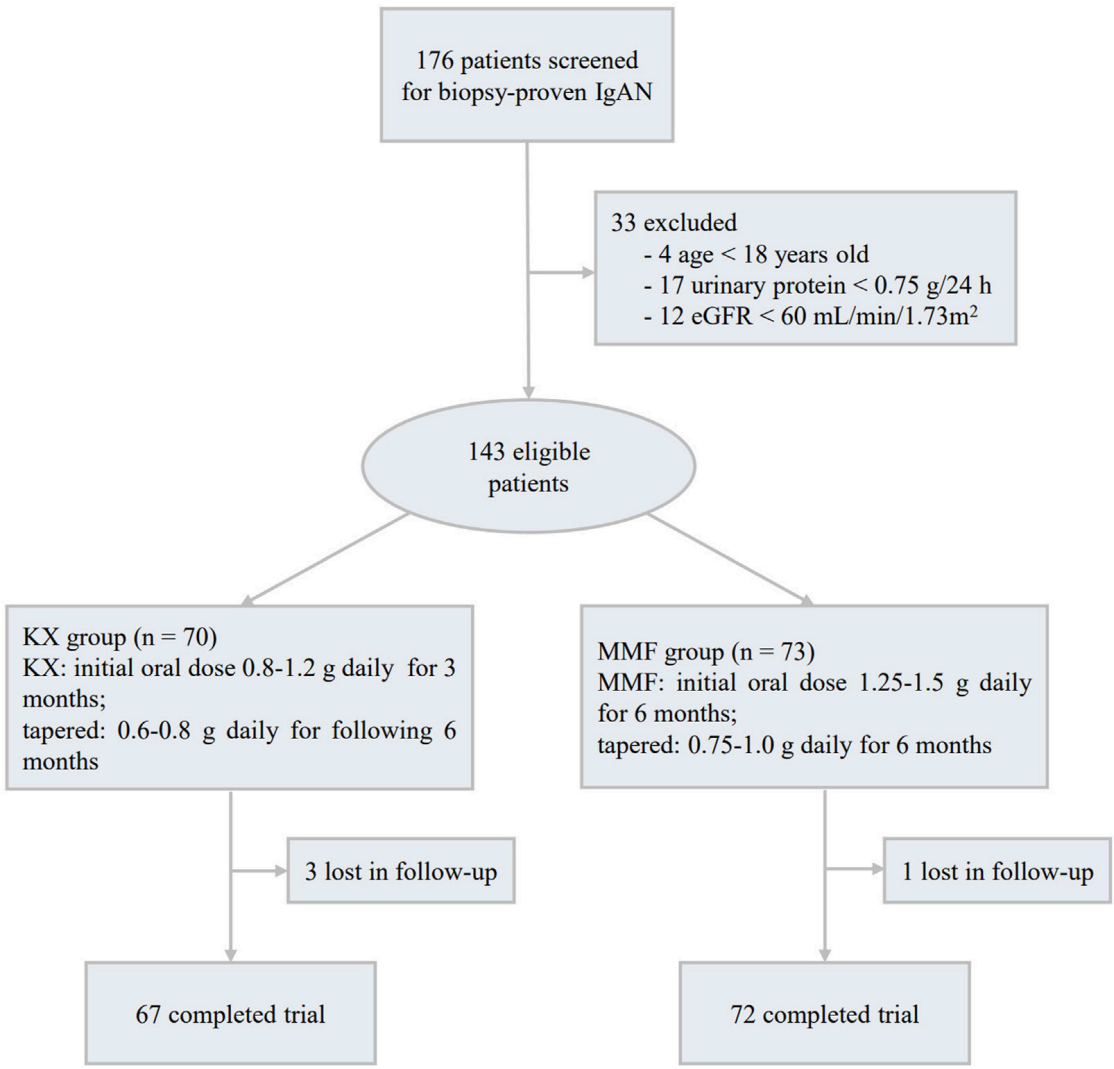
Flow diagram for inclusion of participants.

A total of 176 patients with biopsy-proven primary IgAN were screened, among them, 4 cases were under 18 years old, 17 patients had UP < 0.75 g/d, and 12 patients had an eGFR less than 60 mL/min/1.73 m^2^, these patients were excluded. Among the remaining 143 subjects, 70 patients were assigned to receive KX treatment and 73 to receive MMF,4 patients (3 patients in the KX group and 1 in the MMF group) were lost to follow-up, and 139 patients (97.2%) completed the trial (Figure 1). The baseline features were comparable between the two groups (*P* > 0.05), and the detailed information is shown in Table 1. The mean (SD) eGFR was 87.75 (15.94) mL/min/1.73 m^2^, and the mean (SD) UP was 1.70 (0.74) g/d.

**TABLE 1:** Characteristics of participants at baseline.

Characteristics	KX group (n = 67)	MMF group (n = 72)	*P*
Age (y)	38.25 ± 10.65	39.24 ± 10.11	0.604
Men (%)	38 (56.7)	45 (62.5)	0.495
Systolic blood pressure (mmHg)	118.63 ± 11.24	117.79 ± 12.45	0.634
Diastolic blood pressure (mmHg)	78.40 ± 10.22	77.28 ± 10.07	0.434
Serum albumin (g/L)	39.93 ± 3.77	40.94 ± 3.87	0.125
Total cholesterol (mmol/L)	4.86 ± 0.80	4.94 ± 0.88	0.784
Triglyceride (mmol/L)	1.74 ± 1.10	1.93 ± 1.31	0.497
LDL cholesterol (mmol/L)	3.08 ± 1.03	3.01 ± 1.39	0.706
HDL cholesterol (mmol/L)	1.31 ± 0.50	1.21 ± 0.56	0.248
Alanine aminotransferase (U/L)	24.31 ± 12.51	25.13 ± 12.23	0.683
Aspartate aminotransferase (U/L)	25.24 ± 6.85	26.35 ± 7.35	0.435
Urine protein (g/24 h)	1.69 ± 0.79	1.71 ± 0.71	0.736
eGFR (mL/min/1.73 m^2^)	89.11 ± 17.39	86.49 ± 14.46	0.448
Pathologic^a^, n (%)			
M1	67 (100.0)	72 (100.0)	—
E1	6 (9.0)	10 (13.9)	0.432
S1	59 (88.1)	58 (80.6)	0.253
T1	26 (38.8)	31 (43.1)	0.730
C1	23 (34.3)	30 (41.7)	0.388

eGFR, estimated glomerular filtration rate; HDL, high density lipoprotein; KX, kunxian; LDL, low density lipoprotein; MMF, mycophenolate mofetil.

^a^ Kidney histologic lesions were graded according to Oxford classification.

### 3.2 Primary outcomes

**FIGURE 2:**
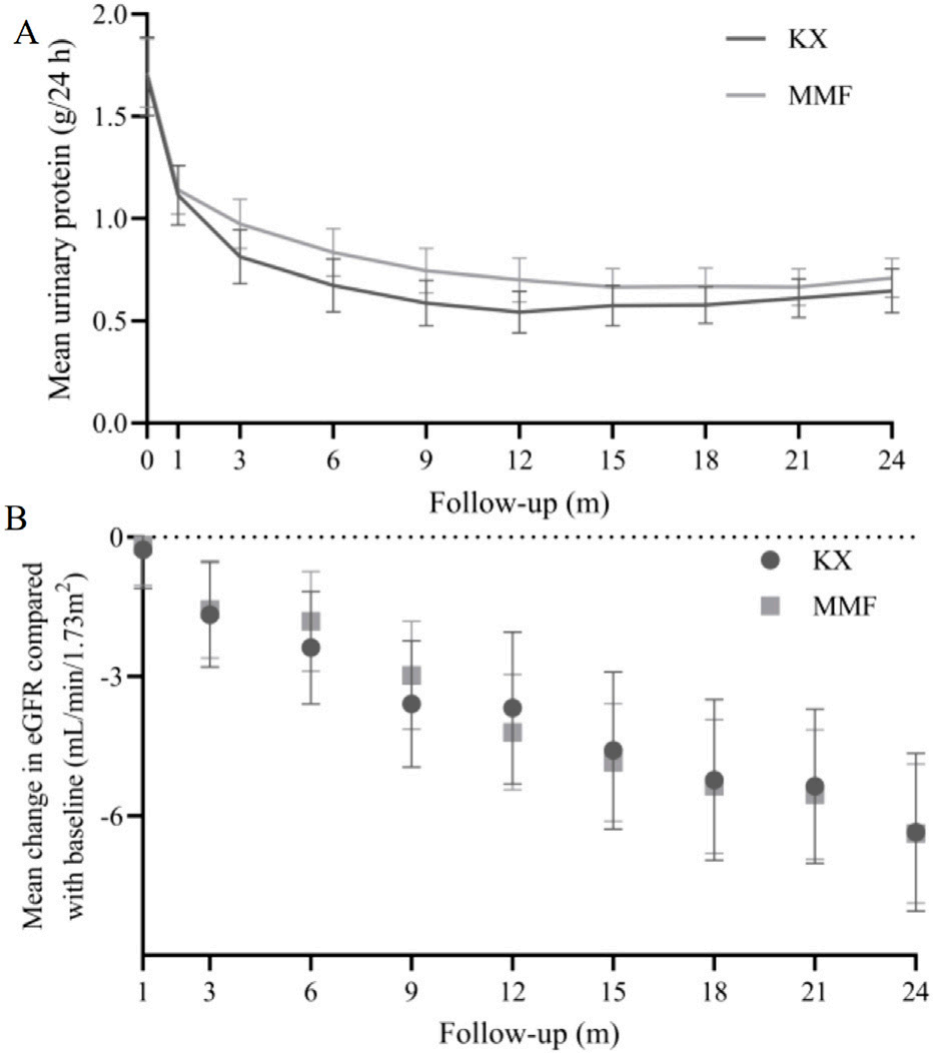
24 h urinary protein **(A)** and eGFR **(B)** change in Kunxian group and mycophenolate mofetil group during 24 months.

Compared with those at the corresponding baseline, there were significantly lower UP levels were found at each of the time points in both groups (Figure 2A). The 24 h UP level in the KX group was significantly lower than that in the MMF group at 3, 6, and 9 months (*P* = 0.046, *P* = 0.008, and *P* = 0.012, respectively). The mean 24 h UP in the KX group versus MMF group at 24 months was very similar to that observed at 9-month. The mean change in eGFR from baseline at each follow-up visit was presented in Figure 2B, and no statistical difference was observed between the two groups.

**FIGURE 3:**
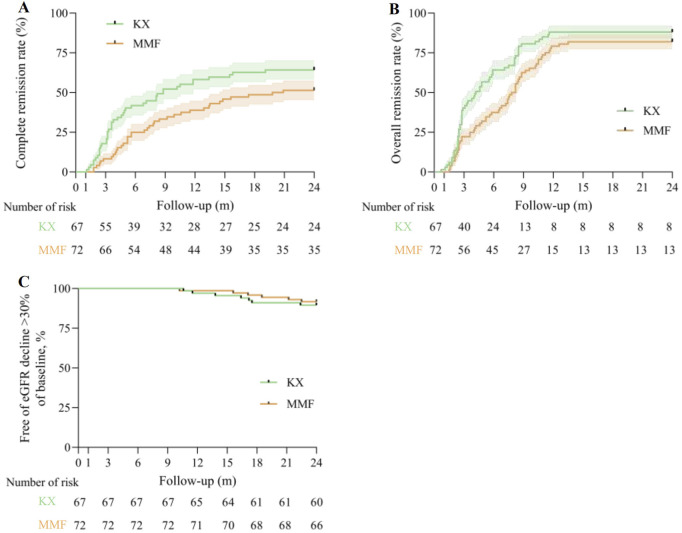
Primary outcomes. **(A)**, Kaplan-Meier analysis for the complete remission rate between two group, log-rank *P* = 0.043. **(B)**, Kaplan-Meier analysis for the overall remission rate between two group, log-rank *P* = 0.025. **(C)**, Kaplan-Meier estimates of the percentage of patients without an eGFR decline of more than 30% of baseline are presented, log-rank *P* = 0.66. eGFR: estimated glomerular filtration rate; KX: Kunxian; MMF, mycophenolate mofetil.

43 patients (64.2%) in the KX group and 37 patients (51.4%) in the MMF group achieved CR at 24 months (log-rank *P* = 0.043, Figure 3A). Similarly, OR occurred in 59 participants (88.1%) in the KX group and 59 participants (81.9%) in the MMF group (log-rank *P* = 0.025, Figure 3B). Compared with MMF, KX treatment increased the rate of the CR and OR by 38.8% (hazard ratio [HR], 0.612, 95% CI 0.385–0.972) and 34.2% (HR 0.658, 95% CI 0.447–0.970), respectively. The percentage of patients without an eGFR decline exceeding 30% of their baseline in both groups was presented in Figure 3C. Specifically, there were 7 patients (10.4%) in the KX group and 6 patients (8.3%) in the MMF group who exhibited a reduction in eGFR of 30% or more. However, no statistically significant difference was observed between the two groups (log-rank *P* = 0.66).

### 3.3 Secondary outcomes

The changes in urine occult blood and URBC between the two groups at baseline and at 6 and 12 months after administration were shown in Table 2. There was no difference in urine occult blood before and after treatment in either group. After 6 months of treatment, the mean (SD) UBCR in the KX group and MMF group was 26.66 (26.77)/uL and 33.74 (25.83)/uL, respectively, and the difference in the mean URBCs between the two groups was significant at this time point (*P* = 0.029). However, at 12 months, no significant difference was noted between the groups (*P* = 0.070).

**TABLE 2:** Secondary outcomes.

	Baseline		*P*	After treatment at 6 months		*P*	After treatment at 12 months		*P*
	KX group	MMF group		KX group	MMF group		KX group	MMF group	
Urine occult blood, n			0.895			0.073			0.341
0	21 (31.3%)	20 (27.8%)		40 (59.7%)	29 (40.3%)		52 (77.6%)	48 (66.7%)	
1	34 (50.7%)	38 (52.8%)		20 (29.9%)	33 (15.8%)		12 (17.9%)	18 (25.0%)	
2	12 (17.9%)	14 (19.4%)		7 (10.4%)	10 (13.9%)		3 (4.5%)	6 (8.3%)	
Urine red blood cell, (N/uL)	55.82 ± 25.49	56.49 ± 32.14	0.794	26.66 ± 26.77	33.74 ± 25.83	0.029	12.84 ± 19.66	18.79 ± 25.44	0.070

Urine occult blood “(-)” and “±” is 0, “1+“, “2+” is 1, and “3+” is 2.

The incidence of AEs was similar in the KX and MMF groups (8.9% vs. 6.9%). No deaths occurred in either group. Menstrual disorders occurred predominantly in the KX group, with 5 cases experiencing scant menstruation or amenorrhea. One patient in the KX group and three patients in the MMF group presented with gastrointestinal symptoms, including abdominal distension and diarrhea. In addition, there were one case of rash in the KX group and two infections in the MMF group.

## 4 Discussion

The present study, with a large sample size and long follow-up period, is the first controlled trial to evaluate the efficacy of KX in the treatment of IgAN. The results showed that 24 h UP levels in the KX group were significantly lower than MMF at 3, 6, and 9 months after dosing. The CR and OR rates at 24 months in the KX group were greater than those in the MMF group (64.2% vs. 51.4%, 88.1% vs. 81.9%), without an increase in AEs.

As a new preparation of TwHF, KX has powerful functions in immunosuppression, anti-inflammation, and lowering proteinuria. A randomized placebo-controlled clinical trial reported that KX effectively improved symptoms and signs in patients with ankylosing spondylitis (Li et al., 2016). Animal experiments proved that KX reduced the proteinuria in lupus nephritis mice and had a protective effect on renal function (Cheng et al., 2023). Some researchers found that KX significantly reduced serum transforming growth factor-β1 levels in primary membranous nephropathy patients and effectively reduced 24 h UP and blood creatinine (Ma et al., 2023). Le et al. showed that proteinuria dropped rapidly after KX treatment, with 33.3% of IgAN patients achieving complete or partial remission within 5 weeks of treatment (Le et al., 2022). Similarly, the present study showed that KX was superior to MMF in reducing 24 h UP in IgAN.

**FIGURE 4:**
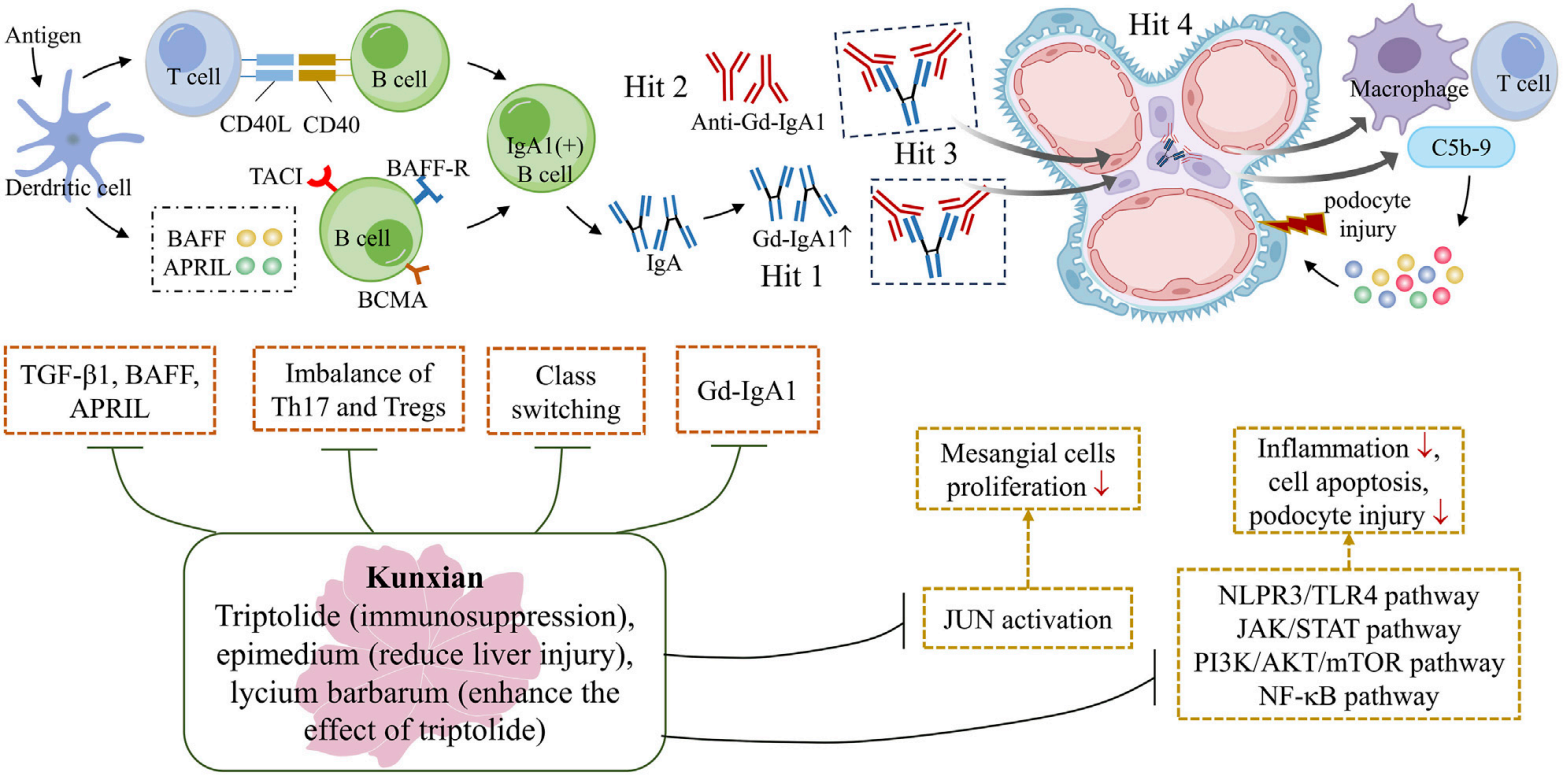
The potential mechanism underlying the protective effect of KX against IgAN. The primary pathogenesis of IgAN is the ‘Multi-hit’ hypothesis, which includes an elevated level of Gd-IgA1, production of anti-Gd-IgA1 autoantibodies, formation of circulating Gd-IgA1 immune complexes and deposition of these complexes on the glomerular mesangium (Stamellou et al. 2023). The triptolide in KX plays a major role in immunosuppression, while Epimedium can reduce liver damage, and Lycium can enhance the effect of triptolide. KX reduces the expression of TGF-β1, BAFF and APRIL, and inhibit IgA class switching, thus reducing the production of pathogenic IgA (Li et al. 2017). It also regulates the immune balance of Th17 and Tregs, slowing down the progression of the disease (Chen et al. 2015). KX inhibits the activation of JUN, a positive regulator of cell proliferation, and thus inhibits the proliferation of mesangial cells (Xia et al. 2021). In addition, KX also modulates multiple signaling pathways, acting as an anti-inflammatory, regulating immune function, promoting apoptosis and attenuating podocyte damage (He et al. 2015). APRIL, a proliferation-inducing ligand; BAFF, B cell-activating factor; KX, Kunxian; Tregs: regulatory T cells; TGF-β1: transforming growth factor-β1.

Overall, accumulated evidence suggests that KX not only reduces the expression of transforming growth factor-β1, a proliferation-inducing ligand and B cell-activating factor, and inhibits IgA class switching, thus reducing the production of pathogenic IgA (Li et al., 2017), but also regulates the balance of Th17 and regulatory T cells in IgAN rats (Chen et al., 2015). Through suppressing multiple signaling pathways, such as the NLPR3/TLR4, JAK/STAT, PI3K/AKT, and NF-κB pathways, KX alleviates kidney inflammation and podocyte damage, thereby reducing proteinuria (Tang et al., 2020; Cheng et al., 2023; He et al., 2015). In addition, KX inhibits the activation of JUN, a positive regulator of cell proliferation, and thus inhibited the proliferation of mesangial cells (Xia et al., 2021), all of which contribute to slowing the progression of the disease in IgAN patients (Figure 4).

It is worth noting that at the end of the observational follow-up at 48 months, 24 h UP in the KX and MMF groups had the same magnitude of effect as observed at 9 months. This finding suggests that the beneficial effects of KX and MMF may not last long after discontinuation. This effect is in line with expectations, as IgAN is a chronic immune-mediated disease, discontinuation of the drug may cause recovery of galactose-deficient IgA1. Therefore, some patients may require long-term low-dose maintenance therapy or re-treatment, further exploration of strategies for appropriate re-treatment is still needed.

Hematuria is a typical presentation of IgAN, persistent hematuria is associated with kidney disease progression (Yu et al., 2020). The present results suggested KX was superior to MMF in reducing UBCR at 6 months post-dosing. The mechanism may be related to the inhibition of mesangial cell proliferation by KX, improvement in the permeability of glomerular filtration membrane, and reduction in red blood cell leakage. However, there is still a lack of research on the mechanism of KX in the treatment of hematuria required further investigation.

In the present study, the rates of AEs were similar in both groups which may be due to the synergistic effect of multiple drugs in KX, Triptolide is the main ingredient, Epimedium and Lycium have hepatoprotective effects, which can enhance the efficacy of the KX while reducing the incidence of AEs (Ma et al., 2023). Menstrual disorder is the most common AEs associated with KX. Of note, most of these AEs cease either spontaneously or after dose adjustments (Wang et al., 2017). To the best of our knowledge, ours was the first study to decrease the KX dose during treatment. Nevertheless, all female patients need to be informed of the potential risk of menstrual disorders before undergoing KX treatment.

Our study had several limitations. First, the present study was not a randomized controlled trial. Second, the long-term clinical outcomes of these patients remain unclear. Finally, the study was conducted in patients in China, and efficacy of KX treatment in other populations is unclear. Therefore, future randomized controlled trials with large samples are needed to confirm the established relationship and to further investigate the long-term prognosis. Taken together, this prospective cohort study firstly indicated that KX was safe and significantly reduced proteinuria in IgAN patients compared with MMF.

## Data Availability

The original contributions presented in the study are included in the article/supplementary material, further inquiries can be directed to the corresponding author.

## Abbreviations

AEs, adverse events; CR, complete remission; eGFR, estimated glomerular filtration rate; IgA, immunoglobulin A; IgAN, Immunoglobulin A nephropathy; KX, Kunxian; MMF, Mycophenolate mofetil; OR, overall response; SD, standard deviation; TwHF, Wilfordii Hook F; UP, urinary protein; URBCs, urine red blood cells.

## References

[B1] Chen F. Ma Y. L. Ding H. Chen B. P. (2015). Effects of Tripterygium wilfordii glycosides on regulatory T cells and Th17 in an IgA nephropathy rat model. Genet. Mol. Res. 14 (4), 14900–14907. 10.4238/2015.November.18.55 26600551

[B2] Chen Y. Gong Z. Chen X. Tang Li Zhao X. Yuan Q. (2013). Tripterygium wilfordii Hook F (a traditional Chinese medicine) for primary nephrotic syndrome. Cochrane Database Syst. Rev. 10.1002/14651858.CD008568.pub2 PMC13034419 23934958

[B3] Chen Y.-Z. Gao Q. Zhao X.-Z. Chen X.-M. Zhang F. Chen J. (2010). Meta-analysis of Tripterygium wilfordii hook F in the immunosuppressive treatment of IgA nephropathy. Intern. Med. 49 (19), 2049–2055. 10.2169/internalmedicine.49.3704 20930429

[B4] Cheng C. Zhu R. Liu M. Yang H. Guo F. Du Q. (2023). Kunxian capsule alleviates renal damage by inhibiting the JAK1/STAT1 pathway in lupus nephritis. J. Ethnopharmacol. 310, 116349. 10.1016/j.jep.2023.116349 36924861

[B5] He L. Peng X. Liu G. Tang C. Liu H. Liu F. (2015). Anti-inflammatory effects of triptolide on IgA nephropathy in rats. Immunopharmacol. Immunotoxicol. 37 (5), 421–427. 10.3109/08923973.2015.1080265 26466641

[B7] Jia Y. Lei L. Luo X. Zhao Z. Wang M. van Andel T. (2021). Analysis of historical changes in traditional Chinese medicine based on an Indonesian collection of Chinese materia medica from c. 1870. J. Ethnopharmacol. 269, 113714. 10.1016/j.jep.2020.113714 33352236

[B8] Jin Bo Liu J. Zhu Y. Lu J. Zhang Q. Liang Y. (2023). Kunxian capsule alleviates podocyte injury and proteinuria by inactivating β-catenin in db/db mice. Front. Med. 10, 1213191. 10.3389/fmed.2023.1213191 PMC10349331 37457567

[B9] Le W.-Bo Shi J.-S. Gong S.-W. Yang F. (2022). Effectiveness and safety of KunXian capsule for the treatment of IgA nephropathy. BMC Nephrol. 23 (1), 179. 10.1186/s12882-022-02814-7 35538439 PMC9088128

[B10] Levey A. S. Stevens L. A. Schmid C. H. Zhang Y. L. Castro A. F. 3rd Feldman H. I. (2009). A new equation to estimate glomerular filtration rate. Ann. Intern Med. 150 (9), 604–612. 10.7326/0003-4819-150-9-200905050-00006 19414839 PMC2763564

[B11] Li H. Kong D. Xu Y. Li X. Yao G. Chen K. (2017). Tripterygium Wilfordii inhibits tonsillar IgA production by downregulating IgA class switching in IgA nephropathy. Oncotarget 8 (65), 109027–109042. 10.18632/oncotarget.22561 29312588 PMC5752501

[B12] Li L.-S. Liu Z.-H. (2004). Epidemiologic data of renal diseases from a single unit in China: analysis based on 13,519 renal biopsies. Kidney Int. 66 (3), 920–923. 10.1111/j.1523-1755.2004.00837.x 15327382

[B13] Li Q. Li Li Bi L. Xiao C. Lin Z. Cao S. (2016). Kunxian capsules in the treatment of patients with ankylosing spondylitis: a randomized placebo-controlled clinical trial. Trials 17 (1), 337. 10.1186/s13063-016-1438-6 27449221 PMC4957347

[B14] Ma R. Kannan M. Zhuang K. Xia Q. Sun D. Tu P. (2023). Pharmacological importance of Kunxian Capsule in clinical applications and its adverse effects: a review. Chin. Herb. Med. 15 (2), 222–230. 10.1016/j.chmed.2022.08.011 37265775 PMC10230640

[B15] Qin Y. Jin Z. Xiao W. Wang Y. Yu Z. Zhang Y. (2023). Distribution of pathological types and epidemiological characteristics based on kidney biopsy in Northwest China. Kidney Res. Clin. Pract. 42 (1), 63–74. 10.23876/j.krcp.21.296 36328996 PMC9902739

[B16] Shan Yu Zhao J. Wei K. Jiang P. Xu L. Chang C. (2023). A comprehensive review of Tripterygium wilfordii hook. f. in the treatment of rheumatic and autoimmune diseases: bioactive compounds, mechanisms of action, and future directions. Front. Pharmacol. 14, 1282610. 10.3389/fphar.2023.1282610 38027004 PMC10646552

[B17] Stamellou E. Seikrit C. Tang S. C. W. Boor P. Tesař V. Floege J. (2023). IgA nephropathy. Nat. Rev. Dis. Prim. 9 (1), 67. 10.1038/s41572-023-00476-9 38036542

[B18] Tang Y. Zhang Yi Lin Li Xie Z. Wen C. Huang L. (2020). Kunxian capsule for rheumatoid arthritis: inhibition of inflammatory network and reducing adverse reactions through drug matching. Front. Pharmacol. 11, 485. 10.3389/fphar.2020.00485 32362827 PMC7181472

[B19] Wang Z. Yu C. Zhou L. N. Chen X. (2017). Effects of Tripterygium wilfordii induction therapy to IgA nephropathy patients with heavy proteinuria. Biol. Pharm. Bull. 40 (11), 1833–1838. 10.1248/bpb.b17-00134 28867717

[B20] Xia M. Liu Di Liu H. Zhao J. Tang C. Chen G. (2021). Based on network pharmacology tools to investigate the mechanism of Tripterygium wilfordii against IgA nephropathy. Front. Med. 8, 794962. 10.3389/fmed.2021.794962 PMC8715946 34977095

[B21] Yu G. Z. Guo L. Dong J. F. Shi S. F. Liu L. J. Wang J. W. (2020). Persistent hematuria and kidney disease progression in IgA nephropathy: a cohort study. Am. J. Kidney Dis. 76 (1), 90–99. 10.1053/j.ajkd.2019.11.008 32197881

[B22] Zhang X. Xia J. Jiang Y. Pisetsky D. S. Smolen J. S. Mu R. (2024). 2023 international consensus guidance for the use of Tripterygium wilfordii hook F in the treatment of active rheumatoid arthritis. J. Autoimmun. 142, 103148. 10.1016/j.jaut.2023.103148 37967495

